# The Role of Reduced Graphene Oxide in the Suspension Polymerization of Styrene and Its Effect on the Morphology and Thermal Properties of the Polystyrene/rGO Nanocomposites

**DOI:** 10.3390/polym12071468

**Published:** 2020-06-30

**Authors:** Marta Sieradzka, Janusz Fabia, Dorota Biniaś, Ryszard Fryczkowski, Jarosław Janicki

**Affiliations:** Institute of Textile Engineering and Polymer Materials, University of Bielsko-Biala, 43-309 Bielsko-Biala, Poland; jfabia@ath.bielsko.pl (J.F.); dbinias@ath.bielsko.pl (D.B.); rfryczkowski@ath.bielsko.pl (R.F.); jjanicki@ath.bielsko.pl (J.J.)

**Keywords:** in situ polymerization, reduced graphene oxide, polystyrene beads, morphology of beads

## Abstract

Reduced graphene oxide (rGO) was used to obtain Polystyrene (PS)/rGO nanocomposites via in-situ suspension polymerization. The main goal of the article was to determine how rGO influences the morphology and thermal properties of PS beads. The obtained samples were studied by means of a scanning electron microscope (SEM), and calorimetric and thermogravimetric analysis (DCS, TGA). It was proven that the addition of rGO, due to the presence of polar functional groups, causes significant changes in bead sizes and size distribution, and in their morphology (on the surface and in cross-section). The increasing amount of rGO in the polymer matrix increased the size of beads from 0.36 to 3.17 mm for pure PS and PS with 0.2 wt% rGO content, respectively. PS/rGO nanocomposites are characterized by distinctly improved thermostability, which is primarily expressed in the increase in their decomposition temperature. For a sample containing 0.3 wt% rGO, the difference is more than 12 °C in comparison to pure PS beads.

## 1. Introduction

Nanocomposites with the addition of various allotropic forms of carbon, such as carbon black, carbon nanotubes or fullerene, have been investigated for many years [1,2,3,4]. The discovery of graphene, which is characterized by excellent mechanical, thermal and electrical properties, has resulted in numerous attempts to apply it in many fields in our daily life. For this reason, intensive research of nanocomposites with graphene (and/or its derivatives) is carried out to obtain a new class of materials that could be applied as biosensors, supercapacitors, solar cells or for electromagnetic shielding (EMI) [5,6,7,8]. The mechanical, thermal and electrical properties of graphene-based nanocomposites are influenced not only by the type and quality of components, but also by other factors. The most important of them include: the degree of dispersion of the nanoadditive, the interaction between the polymer matrix and the nanoadditive, the specific surface area and the size of the filler in relation to the polymer matrix [9]. Nevertheless, the method of obtaining nanocomposite has also a significant influence on its properties. Of course, there are many different methods that allow the production of nanocomposites, including those containing graphene, but the most commonly used methods include: in-situ polymerization, solvent processing, melt blending and layer by layer assembly [9,10,11,12]. The first of these methods consists in carrying out a polymerization reaction that is initiated in a mixture containing the appropriate monomer (and/or oligomer) and graphene [13]. The nanocomposites prepared in this process are characterized by very good mechanical properties, with a much lower percolation threshold, compared to nanocomposites obtained using other methods. This is due to the good dispersion of the nanoadditive and strong interactions between respective components that are formed during the polymerization reaction [12].

Polystyrene (PS) is a polymer widely used for numerous applications in our daily life for example in architecture, packing, automotive, and many other fields [14,15,16,17]. The best known application of this polymer is expandable polystyrene (EPS), also known under the trade name of Styrofoam. It is a product made from polystyrene beads that is pre-foamed and molded into the desired shaped by high pressure steam [18]. Despite the fact that EPS is an excellent thermal insulator, there are constant attempts to further improve its thermal properties. To that end, during the production process of PS beads, some substances—such as carbon black, graphite, metal oxides, and powdered metals—are introduced [19,20,21]. Wu et al. [22] prepared the aluminum nitride/PS (AlN/PS) composites by the powder processing technique, and investigated the thermal properties of the obtained composites. As the authors report, the thermal stability of the AlN/PS composite was improved with the increasing addition of AlN. The thermal conductive coefficient (λ) of the AlN/PS composites was enhanced from 0.189 to 0.418 W/(m·K) when the content of AlN was 25 wt%. Improvement in the thermal properties of polystyrene was also observed by Yan et al. [23] who obtained composites containing modified carbon black. The authors conclude that the decomposition temperature of the composite was about 22 °C higher than that of pure PS. It seems that graphene, discovered in recent years, could also be considered as an additive improving the thermal properties. H. Hu et al. [24] prepared graphene nanosheets–polystyrene nanocomposites via in-situ emulsion polymerization. The investigation of the glass-transition region of the nanocomposites and the thermal stability demonstrated that the introduction of graphene nanosheets into the polystyrene matrix is very beneficial in the sense of thermal properties improvement of the nanocomposite. Flame-retardant performances of PS nanocomposites were studied by Y. Han et al. [15]. The authors obtained PS nanocomposites with thermally reduced graphene oxides at different reduction temperatures. Visible improvement in flame retardant performance was achieved with the addition of rGO reduced at the highest temperature. The peak release rate reached 862 kW/m^2^ and 452 kW/m^2^ for pure PS and PS/graphene nanocomposite, respectively. The authors explained that it is attributed to the appropriate removal of the oxygen-containing functional groups, as well as the disordered expanded layer structure in reduced graphene oxide.

Suspension polymerization was first developed by Hoffman and Delbruch in 1909 [25], and it is a well-known method of polystyrene production on an industrial scale untill now [26]. This process involves the dispersion of styrene in the presence of the initiator in aqueous phase with the addition of a stabilizer in the water–oil suspension [24]. The stirring of reagents causes the dispersion of small monomer droplets in the water phase. The suspension polymerization process is considered as a case of bulk polymerization. Free-radical polymerization occurs in a drop of monomer, which is suspended in water phase. Each dispersed drop of monomer is a “microblock” from which the heat of polymerization is transferred to the water phase. There are several factors that allow us to keep a balance of the reaction system, for example: suspension agent, rate of stirring, initiator (type, amount), oil/water phase ratio. If some parameter changes, the balance of the reaction system may be upset, and then the process will be difficult to continue [27,28]. The introduction of various types of additives might also cause instability in the polymerization system.

The main goal of the work was to obtain PS/rGO nanocomposites by in-situ suspension polymerization, and determine the role of the nanoadditive in the process. Reduced graphene oxide prepared by low-temperature thermal reduction is a few-layer material, which in its structure has partially unreduced oxygen-containing functional groups. On the one hand, these groups ensure the occurrence of intermolecular interactions between the components; but on the other hand, they might inhibit the polymerization process by reaction with the initiator. We studied the effect of reduced graphene oxide (rGO) addition on the morphology and thermal properties of obtained samples.

## 2. Materials and Methods 

### 2.1. Materials

The reagents for obtaining reduced graphene oxide were as follows: graphite powder <20 µm (Sigma-Aldrich, Poznan, Poland), 98% H_2_SO_4_, KMnO_4_, 30% H_2_O_2_, and 35–38% HCl, were supplied by Chempur S.A. (Piekary Slaskie, Poland). All reagents were used as received, without further purification. The other materials for polymerization, styrene and benzoyl peroxide (BPO), were supplied by Sigma-Aldrich. Gelatine powder was purchased from Chempur S.A. (Poland).

### 2.2. Preparation of Graphene Oxide and Reduced Graphene Oxide

Graphene oxide was prepared from graphite using a modified Hummers method, and reduced graphene oxide was prepared by the low-temperature thermal reduction. Both procedures were described by the authors in earlier publications [29,30], and details are provided in Supplementary Materials.

### 2.3. Preparation of PS/rGO Nanocomposites

The suspension polymerization protocol was as follows: A certain amount of deionized water and gelatine were added into a three-necked flask and heated to 80 °C with stirring. At the same time, styrene was pretreated with sodium hydroxide to remove inhibitors. Purified styrene was mixed with a determined amount of reduced graphene oxide and BPO. When the temperature of the reaction mixture reached 80 °C, the reactant styrene/BPO/rGO was added by portions. A total of five different samples were prepared, varying the weight percentage of reduced graphene oxide (Table 1). The suspension polymerization was carried out for 8 h. The obtained beads were washed with distilled water several times and were dried at 50 °C to constant weight.

An attempt was also made to obtain a nanocomposite containing 0.3 wt% rGO. However, the polymerization process did not succeed when the same proportion of reagents was maintained (Figure S1a). Obtaining the PS-0.3 nanocomposite was only possible after modification of the reagent composition, which is listed in Table 1.

### 2.4. Methods

The morphology of the samples was observed using a Phenom scanning electron microscope (SEM). The diameters of the beads were determined using the Phenom software. For each type of bead, 50 measurements of diameters were performed, and then the distribution and mean values were determined. Phenom ProX scanning microscope equipped with an energy-dispersive spectrometer (EDS) was used to analyse the chemical composition of the obtained graphene oxide and reduced graphene oxide.

Wide-angle X-ray scattering (WAXS) analyses were carried out using a URD 63 Seifert diffractometer. CuKα radiation at an accelerating voltage of 40 kV and an anode current intensity of 30 mA was used. Measurements were performed in the range of angles from 5° to 55°, with increments of 0.1° for a range of 5–38°, and of 0.05° for a range of 38.05–55°.

Fourier Transform Infrared Spectroscopy (FTIR) measurements were taken using a Nicolet 6700 FT-IR spectrometer (Thermo Electron Corp., Madison, WI, USA) equipped with photoacoustics MTEC model 300 accessory. The following measurement parameters were used: resolution, 8 cm^−1^; spectral range, 500–4000 cm^−1^; (DTGS) detector; number of scans, 64. Data collection and post-processing were performed using OMNIC software (v. 8.0, Thermo Electron Corp.).

Differential scanning calorimetry (DSC) was performed using a TA Instruments Thermal Analysis System 5100 equipped with TA Instruments 2920 Calorimeter and RCS cooling system. The temperature was calibrated with the melting point of indium (156.6 °C) and the enthalpy was calibrated with indium (28.4 J/g). The samples were heated from 0 to 210 °C with the heating rate of 20 °C/min under nitrogen atmosphere (flow rate of 40 mL/min).

Thermogravimetric analysis (TGA) was performed using a Thermogravimetric Analyzer Q500 TA Instruments. Measurements were carried out in a temperature range from 30 to 650 °C with the heating rate of 20 °/min in nitrogen gas (purge 60 mL/min). Pre-tared platinum pans were used to contain approx. 20 mg.

The DSC and TGA data were evaluated by means of the Universal V4.5A (TA Instruments) software. 

## 3. Results and Discussion

### 3.1. Characterisation of Reduced Graphene Oxide

Before the introduction of rGO into the polymer matrix, the nanoadditive was characterized by the WAXS, TGA, EDS and FTIR studies. 

**Figure 1:**
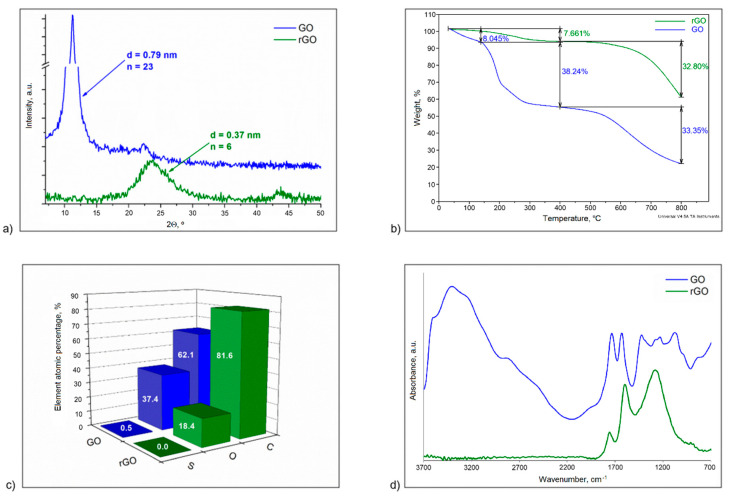
Characterisation of graphene oxide and reduced graphene oxide: (**a**) WAXS patterns (d—interlayer distance, n—average number of layer); (**b**) TG curves; (**c**) EDS analysis; (**d**) FTIR spectra.

Figure 1a shows WAXS patterns of graphene oxide and reduced graphene oxide. Graphene oxide is characterized by sharp peak located at an angle of 2Θ = 11.2°. Formation of oxygen-containing functional groups on the surface of the graphite layers caused the distance between the layers to increase from 0.33 to 0.79 nm for graphite and GO, respectively. The consequence of the oxidation process is also a change in the size of the crystallites, the average size of which in this case is 17.5 nm. As a result of thermal reduction, the characteristic peak of graphene oxide vanishes, while a broad and low intensity peak appears at an angle of 2Θ = 23.9°. This shift is associated with a reduction in the interlayer distance in reduced graphene oxide to 0.37 nm, which occurred as a result of the removal of oxygen-containing functional groups [31]. The broad peak of rGO indicates that less crystalline regions can be observed in this structure in comparison to graphite or graphene oxide [32]. During reduction, the graphite structure is not restored, and random and disordered restoration of the layered structure results in the occurrence of much smaller crystallites. Nevertheless, the oxidation and reduction processes decrease the number of layers, and the average number of layers (n) in rGO is 6, as shown in Figure 1a.

The three-step weight loss is observed on the thermogram for graphene oxide in Figure 1b. The first stage of weight loss (approximately 8%) is associated with the removal of adsorbed water. The next two of steps are associated with the removal of oxygen-containing functional groups present in the GO structure. The second shift of TG curve is caused by the decomposition of less stable oxygen-containing functional groups (e.g., hydroxyl, epoxy groups) and occurs at a temperature range of 150–400 °C. Above 400 °C, a third step of weight loss is observed where more stable oxygen-containing functional groups, e.g., carbonyl and carboxylic groups, are eliminated. In the case of reduced graphene oxide, only the two-step weight loss is observed. At temperatures up to 400 °C, the sample loses only about 8% of its weight. This means that low-temperature thermal reduction is an efficient way to remove less stable oxygen-containing functional groups from graphene oxide. The above results were confirmed by EDS analysis (Figure 1c). As a result of the removal of functional groups, the amount of oxygen in rGO was reduced by half.

Figure 1d shows the FTIR spectra, in which the characteristic bands for GO and rGO are visible. The significant changes are observed in the bands at 3700-2200 cm^−1^ and in the fingerprint region (1500–400 cm^−1^), where the bands characteristic for oscillators of less stable oxygen-functional groups are visible. Although the thermal reduction was carried out, the characteristic bands for carbonyl and carboxyl groups at 1745 cm^−1^ and 1290 cm^−1^ and the band at 1622 cm^−1^ corresponding to the aromatic C=C ring stretching can still be found. 

Reduced graphene oxide, with the parameters described above, was used in the suspension polymerization process of styrene.

### 3.2. Characterisation of PS/rGO Nanocomposites

**Figure 2:**
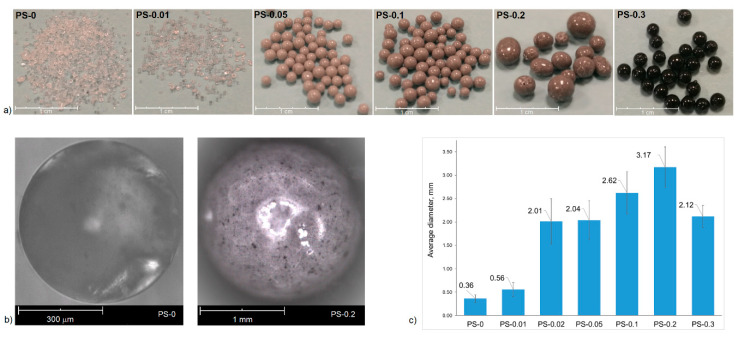
Characterisation of pure PS and PS/rGO beads: (**a**) digital photos; (**b**) optical microscope images of PS-0 and PS-0.2 beads; (**c**) average diameters of beads size.

Beads characteristic for suspension polymerization were obtained in this work. As shown in Figure 2, the samples differ in both colour (a) and size (c).

**Figure 3:**
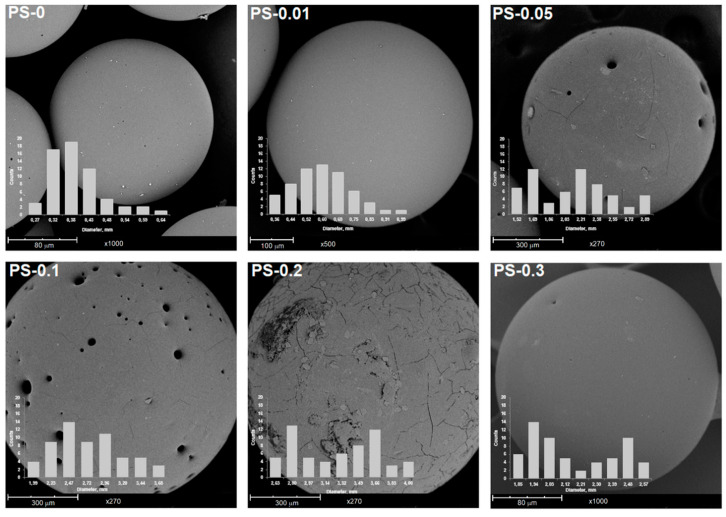
SEM images of pure PS and PS/rGO nanocomposites with particles size distribution.

In Figure 2a, it can be observed that PS-0 and PS-0.01 beads are transparent, but an increasing amount of reduced graphene oxide caused the light transmittance of the beads to decrease. The increase in the content of rGO, which is a black powder, caused the colour of the beads to change from light grey to black. It can be also seen that the reduced graphene oxide is distributed uniformly over the surface of the spheres without creating clearly visible agglomerates (Figure 2b). The results show that an increase in the rGO content in the polymer matrix caused a growth of the beads diameter from 0.36 to 3.17 mm for PS-0 and PS-0.2, respectively (Figure 2c). It is well-known that the size of the beads obtained during suspension polymerization depends on many factors, such as: speed of stirring, geometry of the stirrer and reactor, surface tension, monomer viscosity, etc. [33,34,35]. In this case, when the other factors were not modified, the most important parameter is the change of the surface tension at the oil–water interface of the suspension, which is connected with using of the reduced graphene oxide. During suspension polymerization, suspension stabilizing agents are used to prevent bead agglomeration. The mechanism of action of stabilizers consists in the creation of a thin protective layer on the surface of the monomer drop. Reduced graphene oxide which is dispersed in styrene can adsorb water molecules that reduce surface tension and increase emulsion droplets. The adsorption of water by rGO is possible due to the presence of polar functional groups in its structure. The consequence of this is the growing of the beads’ diameter with the increase in additive amount. The average diameter of PS-0.3, in turn, is smaller compared to PS-0.2, which is associated with a change not only in the amount of rGO, but also the other components of the reaction mixture (Table 1). The polystyrene/graphite beads were obtained by C. Zhang et al. [28]. The authors also observed an increase in the particle size with an increase in graphite content. They elucidated that the viscosity of the monomer dispersion increased with the increasing graphite content, which led to larger size beads. Nevertheless, graphite is a hydrophobic material, and although the results concerning beads size are similar, the mechanism of action of these additives can be different. The morphology of the obtained beads was also observed by SEM (Figure 3). A growing amount of rGO in the polymer matrix caused the surface of the beads to become increasingly defective, with clearly visible holes and microcracks. The reason for the appearance of the defects might be the bigger bead size. The data presented in Figure 2c show that the average size of the PS-0.05 beads is up to 3.6 times larger than that of PS-0.01. Nevertheless, in any sample, no additive coming out of the spheres or adsorbed on their surface was observed. A significant difference is visible for PS-0.3, where the surface is again smooth, practically like for PS-0 and PS-0.01. The use of PS and other proportions in the polymerization system resulted in the improved morphology of the obtained PS-0.3 beads. Reduced graphene oxide also influenced the distribution of diameters of obtained beads, as is presented on the histograms. In all cases where nanocomposites content is not less than 0.05 wt% rGO, the diameter distribution is bimodal. As mentioned, the addition of reduced graphene oxide caused a change in surface tension in the reaction system. The presence of two fractions of beads for these samples may suggest that the amount of suspension stabilizer (gelatine) is not sufficient in relation to the increasing amount of rGO.

**Figure 4:**
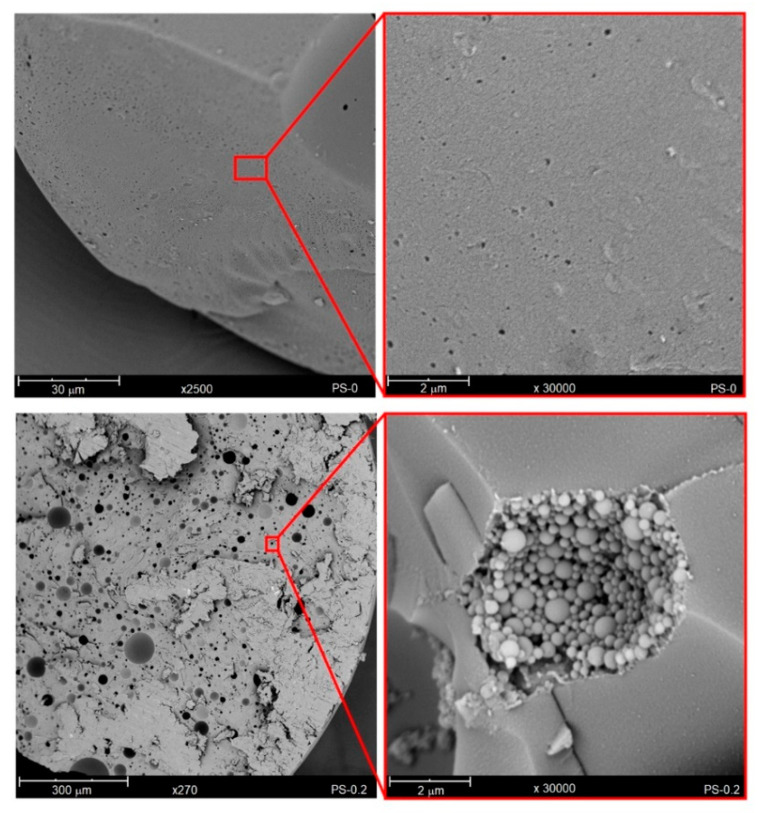
SEM images of cross-section of PS-0 and PS-0.2 nanocomposites.

Figure 4 shows SEM images of cross-sections of PS-0 and PS-0.2 beads. The cross-section of pure PS is smooth and homogeneous, in contrast to the nanocomposite containing 0.2 wt% rGO, which has porous morphology. Interestingly, by increasing the magnification, it could be noticed that nano-beads are present inside the pores. The occurrence of such structures might be related to the process of suspension polymerization. During typical polymerization, the liquid monomer suspended in the form of droplets in the aqueous phase changes into solid polymer particles through the gel stage. The gelled particles have a tendency to attach to each other, e.g., during collision [33], leading to a homogeneous structure represented by the PS-0 sample. The presence of the nano-beads inside the pores, in the case of the PS-0.2, might suggest that at the gelation stage, the spheres are not permanently attached to each other. This may mean that rGO acts as an “internal surfactant”, preventing the formation of a compact structure. This phenomenon was also observed during the polymerization of styrene with 0.3 wt% without the addition of PS (Table 1, Figure S1a). In this case, the polymerization process could not be carried out at all. Of course, there are several reasons that could contribute to this effect. We should be aware that reduced graphene oxide has a complex structure, in which there are various oxygen-containing functional groups. The presence of groups such as quinone groups may act as a factor inhibiting the in-situ polymerization process [36]. Nevertheless, as presented in Supplemental Materials, a successful attempt was made to conduct bulk polymerization of styrene with the addition of 0.3 wt% rGO, and then obtaining the PS/rGO nanocomposite (Figure S1b). This confirms that rGO plays an important role in stabilizing the suspension in which polymerization is carried out. Moreover, to the best of our knowledge, the structure of nano-beads in polystyrene has not hitherto been reported.

**Figure 5:**
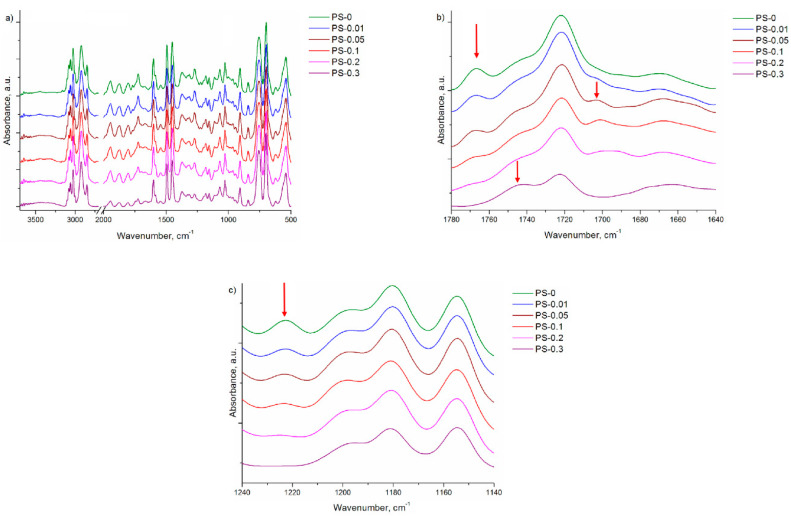
FTIR spectra of PS and PS/rGO beads at different spectral range: (**a**) 3700–500 cm^−1^; (**b**) 1780–1640 cm^−1^; (**c**) 1240–1140 cm^−1^.

FTIR spectra of PS and PS/rGO beads are presented in Figure 5. The pure PS sample is characterized by absorption bands at 3100–3000 cm^−1^ which are assigned to =C–H aromatic stretching vibration and 2922 cm^−1^ which are assigned to –CH_2_ and 2849 cm^−1^ which are assigned to –CH in aliphatic stretching vibration. Three bands with a maximum at 1600 cm^−1^, 1492 cm^−1^ and 1450 cm^−1^ are related to deformation vibration of C–H in benzene ring, and bands at 756 and 699 cm^−1^ are attributed to the C–H out of plane bending vibration of the benzene ring [37]. These bands also appear in all PS/rGO samples. Regions with significant changes are presented in Figure 5b,c. For pure PS beads, there are bands between 1943–1667 cm^−1^ that are assigned to overtones bands in benzene rings [38]. Increasing the amount of rGO in the polymer matrix caused the overlap of the bands at 1763 cm^−1^ and consequently more intensive band is visible at 1743 cm^−1^ for PS-0.3 sample. Another change presented in Figure 5b is associated with the appearance of band at approximately 1700 cm^−1^ for the PS-0.05 sample, widening and shifting towards lower wave numbers with the growing amount of rGO. The differences at 1743 and 1670 cm^−1^ correspond to the characteristic bands of rGO, which are assigned to C=O and C=C (Figure 1d). On the spectrum of the PS-0 sample, a band occurs at a maximum of 1222 cm^−1^, assigned to C–H vibrations in the plane of benzene rings. Increasing the amount of rGO caused this band to initially decrease the intensity, and then for the PS-0.3 sample to disappear completely. Significant changes in the shape of the oscillator bands characteristic of the polystyrene phenyl rings suggest interaction with the graphene rings [39]. The change in oscillation can be the result of the adhesion of both nanocomposite components.

**Figure 6:**
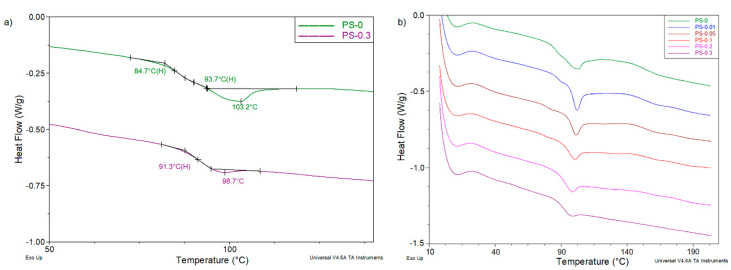
DSC curves registered at the heating mode in the temperature range of the glass transition: (**a**) pure PS and PS-0.3 samples; (**b**) all obtained samples.

Discussion of the thermal investigations begins with the presentation of the results of DSC measurements carried out for all samples at the heating mode in nitrogen, in a rather wide temperature range from 0 to 210 °C using a heating rate of 20 °C/min. Figure 6 shows DSC curves for beads of pure PS and PS/rGO nanocomposites. It should be noted that calorimetric studies reveal the fully amorphous nature of all materials discussed in the paper. Analysis of DSC curves in the temperature range of the glass transition allows us to formulate several important conclusions. For samples containing 0–0.1 wt% rGO, two glass transition temperatures (T_g1_ and T_g2_, respectively) are observed. They are distinctly shifted by 10–18 °C relative to each other (Table 2). The values of both temperatures do not change monotonically as a function of the amount of rGO modifier in the sample, but both the first and second show a similar change tendency. Their highest values (86.5 and 97.1 °C, respectively) were determined for a sample containing only 0.01% of rGO. As the amount of modifier increases, the temperature values T_g1_ and T_g2_ decrease, while the addition of a larger amount of additive to the PS matrix causes only a single shift of DSC curve observed in the glass transition temperature range. As a consequence, one T_g_ value is determined. In this case, the existence of two glass transition temperatures should not be associated with the presence of a heterophasic rGO additive in the polymer (pure PS beads also have two T_g_ values). Most likely, it is a consequence of specific conditions, and more precisely—their change during the formation of the amorphous structure of PS during the polymerization process.

For all tested samples, the glass transition is accompanied by the appearance of a characteristic small endothermic effect on DSC curves. This is the enthalpy relaxation peak, often also referred to as the apparent melting peak (Figure 6) [40]. It reflects the destruction of a certain type of order in the structure of polymer when it is reheated in a calorimeter cell. In no case is it a crystalline order. The discussed order forms spontaneously inside the polymer in the time interval between its fabrication and testing, depending on storage conditions (time and temperature). In the case of the tested samples, the minimum temperature peak of the discussed effect (T_ER_) decreases strictly monotonically as the rGO content increases. The value of the specific enthalpy of this effect (∆H_ER_) changes analogously and decreases more than 10 times (Table 2). It is worth noting that the most drastic change is observed at high levels of rGO.

This clearly indicates that the reduced graphene oxide introduced into the PS structure during the polymerization process, interacting with polystyrene macromolecules, significantly limits their ordering mobility [41,42].

**Figure 7:**
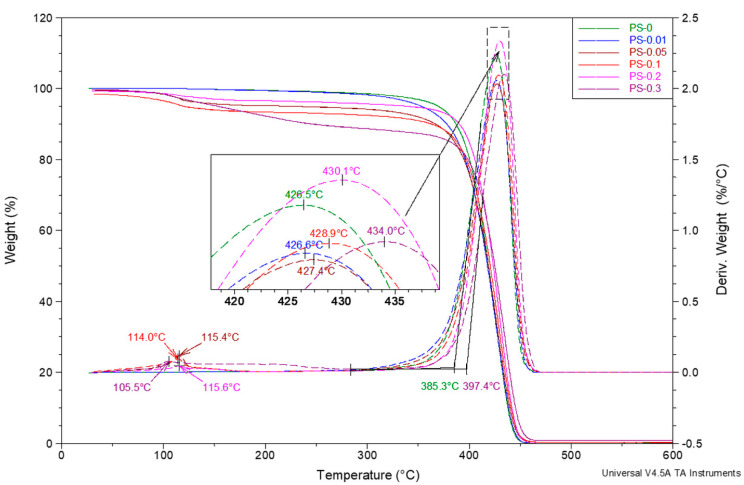
TGA and DTG curves of pure PS and nanocomposite beads with the different content of rGO.

The evaluation of thermal properties of materials produced and described in the paper was continued by conducting thermogravimetric studies. TG and DTG curves of pure PS and PS/rGO nanocomposites are presented in Figure 7. Measurements were carried out up to 600 °C under a nitrogen atmosphere with a heating rate of 20 °C/min. All tested samples are characterized by the occurrence of two types of changes related to mass loss. Considering them in order of increasing temperature, the first one refers to the loss of moisture contained in individual samples, reaching approximately 175 °C. The second one is the thermal dissociation of the base polymer—polystyrene. This change occurs in all cases in one stage in the range of 300-470 °C. With the increase in rGO content, the temperature of the highest decomposition rate, corresponding to the maximum on the DTG curves, shifts quite monotonically upwards. In the extreme case—for a sample containing 0.3 wt% rGO—this difference is 7.5 °C in comparison to pure PS beads. The difference in thermostability (measured by the value of the so-called extrapolated temperature of the beginning of decomposition) is even greater in the discussed case and amounts to over 12 °C. Based on the above results, it can be concluded that a considerable increase in thermal stability can be achieved by the introduction of reduced graphene oxide into the polystyrene matrix [43].

The highest residue after heating to 470 °C is also obtained for the PS-0.3 sample (Table 3). The analysis of the data contained in Table 3 leads to the conclusion that the modification of PS with reduced graphene oxide, with higher rGO content in the matrix (above 0.2 wt%), promotes the formation of condensed carbon structures in the process of thermal dissociation of PS [44,45]. This is expressed as an increase in the amount of residue after heating in nitrogen to 470 °C. It is noteworthy that this increase in residue is much larger than the quantity of the rGO addition.

Based on the TG and DTG curves presented in Figure S7 and the data presented in Table 3, the diverse behaviour of the PS-0.3 sample in the temperature range of 30–280 °C should be noticed. For the PS-0 and PS-0.01 samples, there are no transitions in this range. The others samples in the considered temperature range show only the loss of moisture, which is reflected in the maximum on the DTG curves in a fairly narrow range of 114.0–115.6 °C. However, based on the course of the TG and DTG curves of the PS-0.3 sample, it can be clearly stated that a very pronounced loss of weight in this range (10.66%) has a completely different character. It is most likely caused by the unintentional presence of relatively volatile PS oligomers, not removed from the sample after the polymerization process.

## 4. Conclusions

The article presents the results of research on PS/rGO nanocomposites, which were obtained by in-situ suspension polymerization. It was proved that the chemical structure of reduced graphene oxide plays an important role in the polymerization process, influencing the morphology and thermal properties of PS/rGO samples.

Firstly, the addition of rGO affects the size of the obtained beads. Due to the polar functional groups that are present in the rGO structure, it can adsorb water molecules. This causes a change of surface tension at the oil–water interface in the reaction system. Consequently, this leads to an increase in the diameter of the beads as the content of reduced graphene oxide in the polymer matrix increases. Secondly, during polymerization, the action of rGO can be compared to that of surfactant, which promotes the formation of a porous structure consisting of nano-beads, as shown in the cross-section of the PS-0.2 sample.

The DSC study shows that the addition of reduced graphene oxide during in-situ polymerization influences the amorphous structure of polystyrene, as evidenced by changes in glass transition temperature. Based on the results of thermal investigations, it can be concluded that the modification of PS with rGO through in-situ polymerization leads to a distinct increase in the thermostability of PS/rGO nanocomposites, which is mainly expressed by the increase in their decomposition temperature. This is accompanied by an increase in the residue in the thermal dissociation process and limiting the mobility of the PS matrix to the ordering of the structure.

## Figures and Tables

**Table 1:** Preparation ingredients of pure PS and PS/rGO nanocomposites beads.

	Samples	PS-0	PS-0.01	PS-0.05	PS-0.1	PS-0.2	–	PS-0.3
Water Phase	DW, g	170	170	170	170	170	170	250
	Gel, g	1.7	1.7	1.7	1.7	1.7	1.7	6.3
Oil Phase	St, g	45.4	45.4	45.4	45.4	45.4	45.4	18.0
	PS, g	0	0	0	0	0	0	5.1
	BPO, %	3.0	3.0	3.0	3.0	3.0	3.0	3.5
	rGO, %	0	0.01	0.05	0.1	0.2	0.3	0.3

DW—distilled water, Gel—gelatine, St—styrene, PS—polystyrene, BPO—benzoyl peroxide, rGO—reduced graphene oxide.

**Table 2:** Values of characteristic temperatures of glass transition T_g1_,T_g2_ and enthalpy relaxation peak parameters, T_ER_ and ∆H_ER_, respectively, determined on the basis of DSC measurements.

Sample	*T_g1_*, °C	*T_g2_*, °C	*T_ER_*, °C	*∆H_ER_*, J/g
PS-0	84.7	93.7	103.2	1.77
PS-0.01	86.5	97.1	102.1	1.73
PS-0.05	78.7	95.5	101.3	1.36
PS-0.1	75.8	93.8	100.6	0.69
PS-0.2	–	91.1	98.9	0.56
PS-0.3	–	91.3	98.7	0.17

**Table 3:** Values of weight loss in subsequent thermal transformations of tested samples.

Sample	Weight Loss Up to Temperature 30 °C	Weight Loss in Range of 30–175 °C	Weight Loss in Range of 175–470 °C	Residue after Heating Up to 470 °C
	Δm_1_, %	Δm_2_, %	Δm_3_, %	R, %
PS-0	0.02	0.28	99.70	–
PS-0.01	0.02	0.33	99.65	–
PS-0.05	0.47	4.21	94.97	0.35
PS-0.1	1.47	4.95	93.22	0.36
PS-0.2	0.64	2.67	96.28	0,41
PS-0.3	0.01	10.65 *	88.30	1.04

* for sample PS-0.3 the weight loss up to 280 °C is given.

## Supplementary Materials

The following are available online at https://www.mdpi.com/2073-4360/12/7/1468/s1.
