# Supplementary material for: The Role of Reduced Graphene Oxide in the Suspension Polymerization of Styrene and Its Effect on the Morphology and Thermal Properties of the Polystyrene/rGO Nanocomposites
Source: Polymers (Basel). 2020 Jun 30;12(7):1468. doi: 10.3390/polym12071468 (PMC7407986; doi:10.3390/polym12071468)
Supplement: Supplementary file 1 [file polymers-12-01468-s001.pdf]

polymers

Supplementary Materials

The Role of Reduced Graphene Oxide in the Suspension Polymerization of Styrene and its effect on the Morphology and Thermal Properties of the Polystyrene/rGO Nanocomposites

Marta Sieradzka *, Janusz Fabia, Dorota Biniaś, Ryszard Fryczkowski and Jarosław Janicki

Institute of Textile Engineering and Polymer Materials, University of Bielsko-Biala, Poland, jfabia@ath.bielsko.pl (J.F.); dbinias@ath.bielsko.pl (D.B.); rfryczkowski@ath.bielsko.pl (R.F.); jjanicki@ath.bielsko.pl (J.J.)

* Correspondence: msieradzka@ath.bielsko.pl

Table of content:

S1. Preparation of samples

S2. Optical photographs of samples with 0.3 wt% rGO

S3. The effect of reduced graphene oxide on suspension stabilization

S4. TGA results

S1. Preparation of samples

S1.1. Preparation of graphene oxide

The graphite (3 g) was added into a beaker with 75 cm^3 of sulfuric acid(VI). The mixture was stirred for 1 h at room temperature. Then, the beaker was placed in an ice bath to lower down the temperature of the reaction mixture (below 10 °C). After cooling, $KMnO_4$ was batched into the beaker. After adding the oxidation agent, the mixture remained in the ice-bath for 10 minutes. The oxidation reaction continued for 2.5 h, not exceeding 40 °C. After that time, the sample was washed with distilled water (75 cm^3), warm distilled water (60 °C, 60 cm^3) and 3% of aqueous solution of H_2O_2. The obtained graphene oxide was purified to remove ions coming from the others reagents. For this purpose, GO was washed several times with distilled water and 10% aqueous solution of hydrochloric acid.

S1.2. Preparation of reduced graphene oxide.

The prepared graphene oxide was thermally reduced by micro-explosion. For this purpose, 4 g of partially dried GO was placed in a thermal reduction chamber that was blown with an inert gas (nitrogen). The graphene oxide in the chamber was heated at a rate of approximately 30 °C/min, until micro-explosion occurred. Thermal reduction was carried out until the process ceased to take place in a rapid way. For the obtained graphene oxide, the procedure described above was repeated five times, yielding samples of rGO.

S2. Optical photographs of samples with 0.3 wt% content of rGO

The proportion of reagents are presented in Table S1.

Table S1. Preparation ingredients of PS/rGO nanocomposites with 0.3 wt% rGO content.

	Ingredients	Figure S2a	Figure S2b	Figure S2c
Water phase	Distilled water, g	170	–	250
	Gelatine, g	1.7	–	6.3
Oil phase	Styrene, g	45.4	45.4	18.0
	Polystyrene g	0	0	5.1
	Benzoyl peroxide, %	3.0	3.0	3.5
	Reduced graphene oxide, %	0.3	0.3	0.3

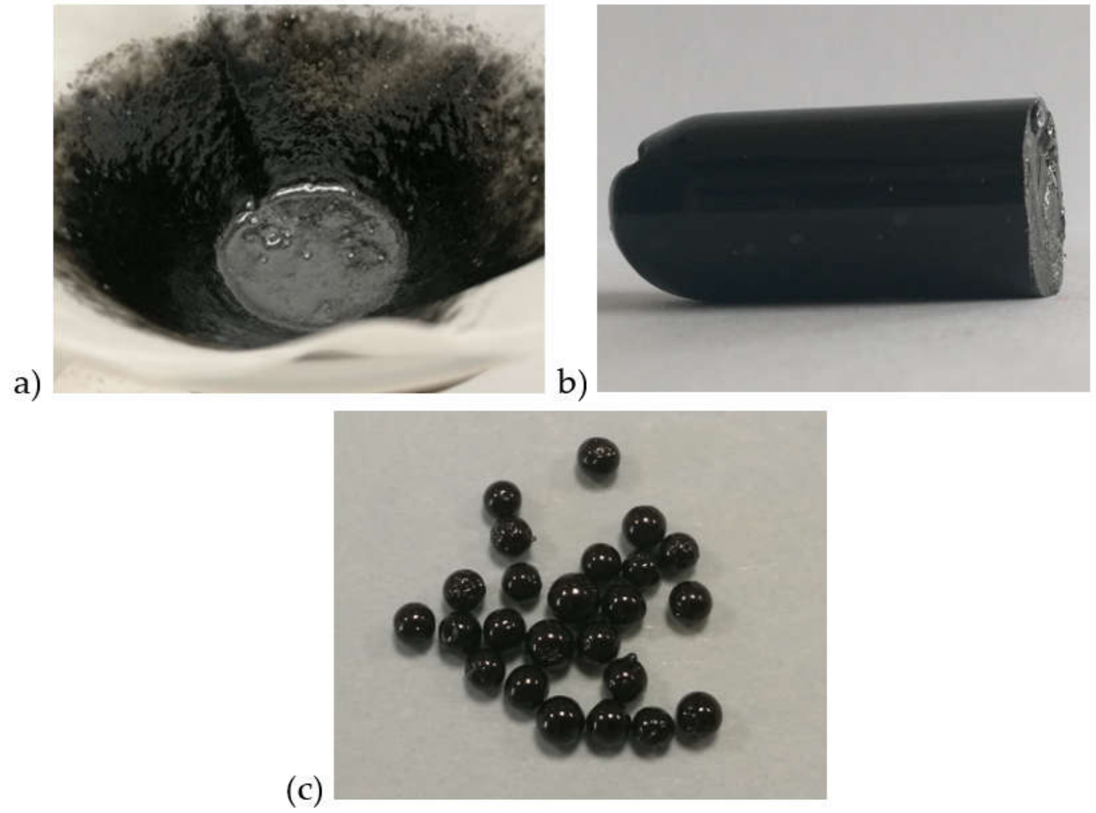

Figure S1. Digital photos of the samples with the same content of rGO obtained by different methods: (**a**) suspension polymerization; (**b**) bulk polymerization; (**c**) suspension polymerization with PS dissolved in styrene.

Suspension polymerization of styrene was described in the article. All processes were carried out under the same conditions (e.g. rate of stirring, time, temperature, composition of the reaction mixture), only the content of reduced graphene oxide varied: 0.01 wt%, 0.05 wt%, 0.1 wt%, up to 0.2 wt%. The addition of 0.3 wt% rGO caused that the suspension polymerization process did not succeed. During this attempt while the reaction mixture was intensively stirred, beads suspended in the water phase were clearly visible. However, after termination of mixing and filtration of resulting mixture, a slime was formed on the filter, as shown in Figure S1a.

The bulk polymerization was carried out to check whether the polymerization process is inhibited by oxygen-containing functional groups present in the structure of reduced graphene oxide. The sample presented in Figure S1b confirms that a nanocomposite containing 0.3 wt% rGO can be obtained. This means that the chemical structure of reduced graphene oxide does not affect bulk polymerization when the amount of nanoadditive is 0.3 wt%, but prevents the suspension polymerization.

In the next study, rGO content was on the same level (0.3 wt%) but the composition of the reaction mixture was significantly changed: polystyrene was dissolved in styrene, and the amount of suspension stabilizer was increased. This caused change in viscosity and surface tension in the reaction system, which consequently allowed to obtain the desirable beads (Figure S1c).

S3. The effect of reduced graphene oxide on suspension stabilization.

In this part, the behaviour of rGO is outlined in a mixture that to some extent simulates the conditions of suspension polymerization. The pictures below present systems containing 0.01 wt% rGO and 0.2 wt% rGO, respectively. These are limiting values where the polymerization took place effectively. For comparison purpose, the samples containing 0.01 wt% and 0.2 wt% graphite were

also prepared, because of this additive is commonly used in the industrial manufacture of polystyrene.

In the first stage of the experiment, four dispersions were prepared: styrene with rGO (0.01 wt% and 0.2 wt%) and styrene with graphite (0.01 wt% and 0.2 wt%). The results directly after sonication and 1 h after sonication are shown in Figure S2 and Figure S3, respectively. In all cases, sedimentation of the additive is visible. However, it has not been observed that sedimentation or formation of agglomerates to be faster for reduced graphene oxide in comparison to graphite, although of presents of oxygen-containing groups in rGO.

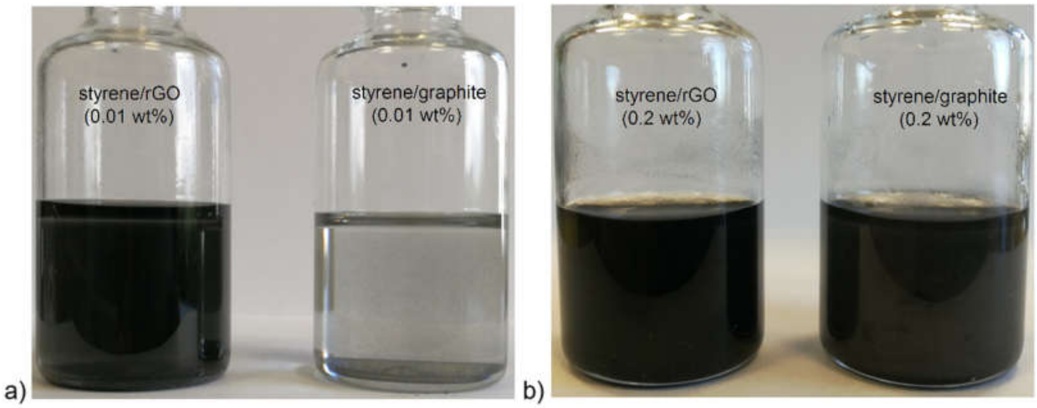

Figure S2. Dispersions of styrene/rGO and styrene/graphite directly after sonication. Content of additives: (**a**) 0.01 wt%; (**b**) 0.2 wt%.

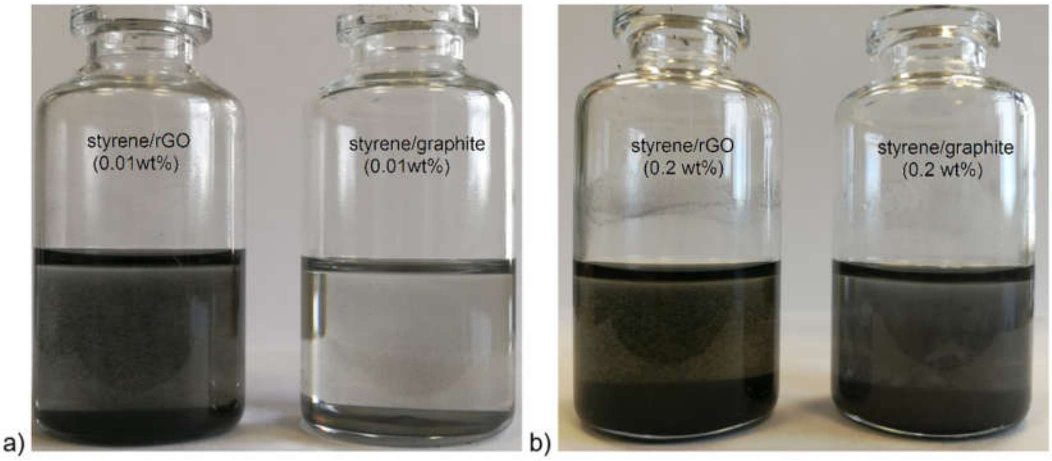

Figure S3. Dispersions of styrene/rGO and styrene/graphite,1 h later. Content of additives: (**a**) 0.01 wt%; (**b**) 0.2 wt%.

Subsequently, distilled water (with the proper amount of gelatine) was added to the styrene/rGO and styrene/graphite dispersions, and the contents of all bottles were shaken vigorously. It can be clearly seen in Figures S4 and S5 that graphite, in all cases, is entirely suspended in styrene. A similar result is also obtained for reduced graphene oxide, but only if its content is 0.01 wt%. When its rising to 0.2 wt%, a significant amount of rGO suspended in the water phase was observed directly after mixing finished. After 1 hour, reduced graphene oxide formed an intermediate layer between oil phase and water phase. This means that reduced graphene oxide plays an important role in suspension polymerization, affecting not only the size of the beads, but also the stabilization of the suspension.

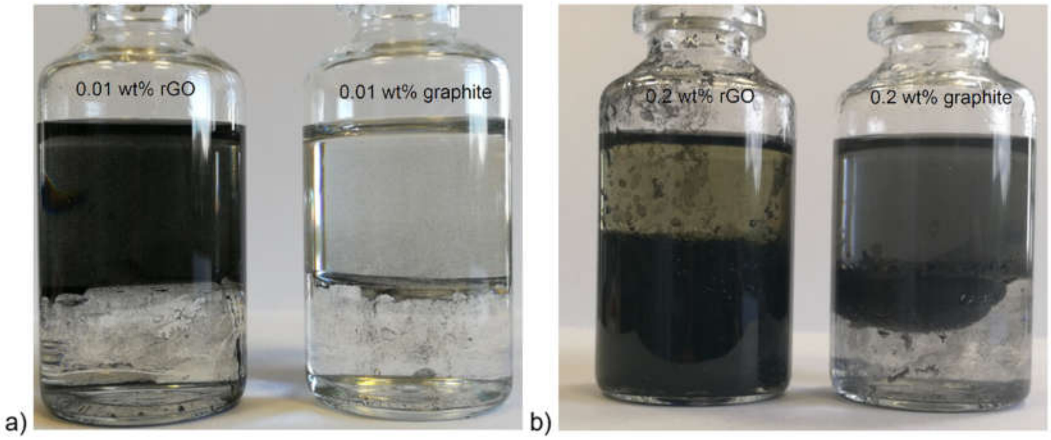

Figure S4. System of oil/water with rGO and graphite a few minutes after mixing. Content of additives: (**a**) 0.01 wt%; (**b**) 0.2 wt%.

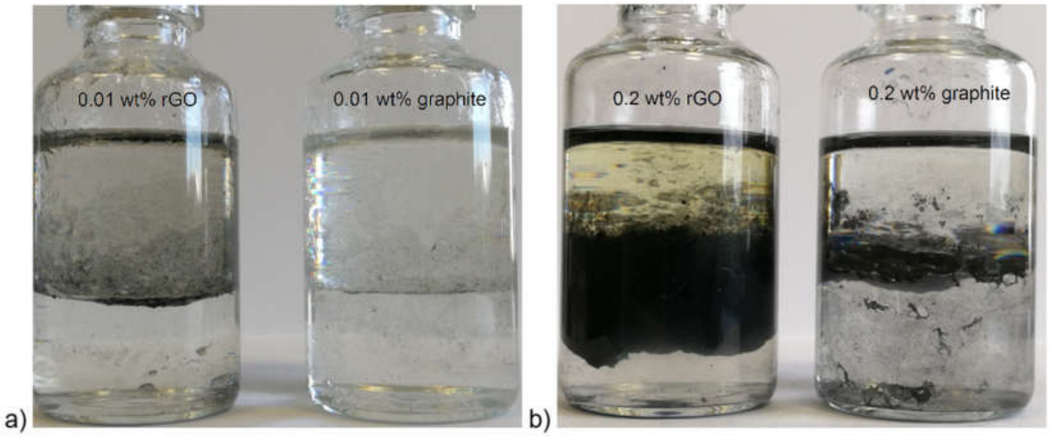

Figure S5. System of oil/water with rGO and graphite, 1 h after mixing. Content of additives: (**a**) 0.01 wt%; (**b**) 0.2 wt%.

S4. TGA results

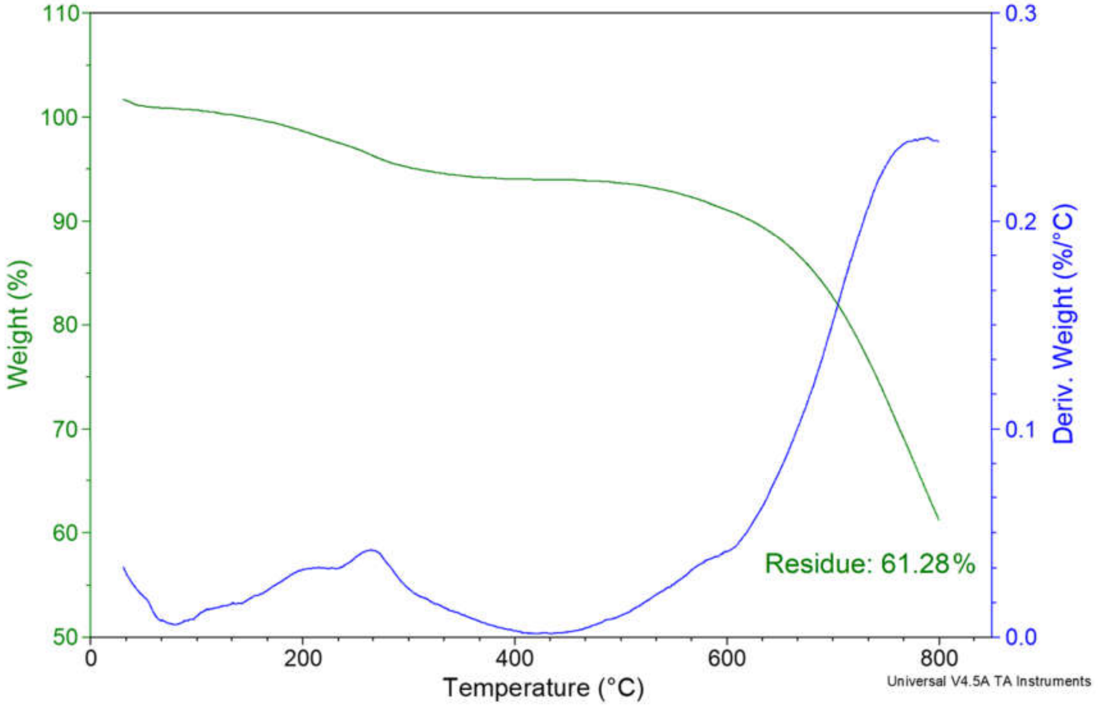

Figure S6. TGA and DTG curves of rGO.

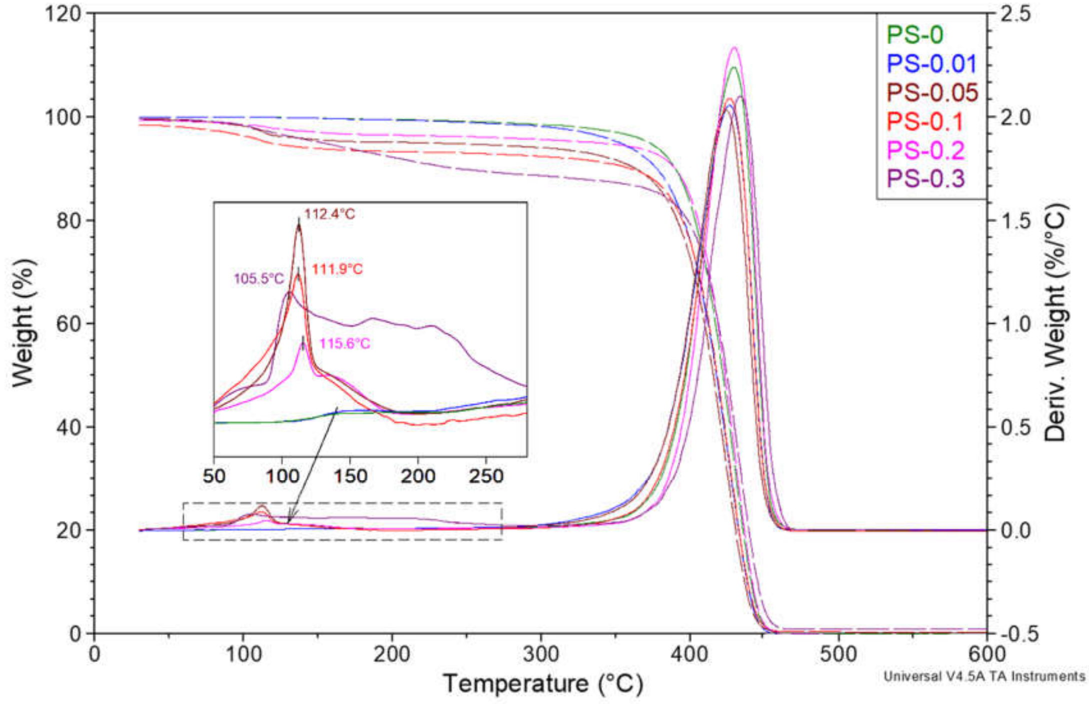

Figure S7. TGA and DTG curves of pure PS and nanocomposite beads with the different content of rGO.

© 2020 by the authors. Submitted for possible open access publication under the terms and conditions of the Creative Commons Attribution (CC BY) license (http://creativecommons.org/licenses/by/4.0/).
